# Aspirin Delimits Platelet Life Span by Proteasomal Inhibition

**DOI:** 10.1371/journal.pone.0105049

**Published:** 2014-08-15

**Authors:** Manasa K. Nayak, Ayusman Dash, Nitesh Singh, Debabrata Dash

**Affiliations:** 1 Department of Biochemistry, Institute of Medical Sciences, Banaras Hindu University, Varanasi, India; 2 Indian Institute of Science Education and Research, Kolkata, India; King's College London School of Medicine, United Kingdom

## Abstract

Aspirin is widely used in clinical settings as an anti-inflammatory and anti-platelet drug due its inhibitory effect on cyclooxygenase activity. Although the drug has long been considered to be an effective and safe therapeutic regime against inflammatory and cardiovascular disorders, consequences of its cyclooxygenase-independent attributes on platelets, the key players in thrombogenesis, beg serious investigation. In this report we explored the effect of aspirin on platelet lifespan in murine model and its possible cytotoxicity against human platelets *in vitro*. Aspirin administration in mice led to significant reduction in half-life of circulating platelets, indicative of enhanced rate of platelet clearance. Aspirin-treated human platelets were found to be phagocytosed more efficiently by macrophages, associated with attenuation in platelet proteasomal activity and upregulation of conformationally active Bax, which were consistent with enhanced platelet apoptosis. Although the dosage of aspirin administered in mice was higher than the therapeutic regimen against cardiovascular events, it is comparable with the recommended anti-inflammatory prescription. Thus, above observations provide cautionary framework to critically re-evaluate prophylactic and therapeutic dosage regime of aspirin in systemic inflammatory as well as cardiovascular ailments.

## Introduction

Although aspirin has been widely used therapeutically against variety of inflammatory conditions since 1890s [1], its anti-platelet activity was not documented until almost 70 years later [2]. The central role of platelets in pathogenesis of occlusive coronary and cerebral thrombotic events has prompted in-depth investigations into molecular underpinnings of aspirin action. Platelets generate thromboxane A2 (TXA2) in response to diverse physiological stimuli like collagen, thrombin and ADP that cause amplification of platelet aggregation and vasoconstriction [3], [4]. Aspirin and other non-steroidal anti-inflammatory drugs (NSAIDs) effectively attenuate activity of the enzyme cyclooxygenase-1 (COX-1), which catalyzes biosynthesis of cyclic prostanoids like TXA2, prostacyclin and other prostaglandins (PG) [5]. Although this accounts for strong anti-thrombotic potential of aspirin, inhibition of prostaglandin synthesis, too, results in altered functions of normally protective prostaglandins with potentially serious consequences. Aspirin-induced inhibition of COX results in loss of cytoprotective effects of PGE2 on gastric mucosa, which partly accounts for gastrointestinal side effects associated with aspirin therapy [5]–[8], especially during high dosage of aspirin administered for treatment of systemic inflammatory ailments including rheumatoid arthritis [9].

Aspirin also elicits effects that are independent of COX inhibition [10]–[17]. Prolonged use of NSAIDs has been reported to reduce the risk of malignancies, though their anti-cancer activity has not been fully established [18]–[22]. Aspirin induces shedding of GPIbα and GPV from platelet surface through activation of the metalloproteinase ADAM17 and cell death [23], [24]. Clinical trials have indicated that chemopreventive properties of NSAIDs could be due to induction of apoptosis [6]–[8]. Several mechanisms have been proposed to explain aspirin-induced apoptosis, which include upregulation of pro-apoptotic proteins [7], [8], [25], [26] and oxidative stress [27], [28]. Aspirin has been shown to induce decrement in mitochondrial transmembrane potential and stimulate intrinsic pathway of apoptosis in mouse Neuro 2a cells [29]. There have been few clinical studies to suggest decrease in platelet count in individuals under aspirin medication [30]–[32]. Although aspirin has long been considered to be an effective and safe therapeutic regime against cardiovascular disorders, consequences of its pro-apoptotic attributes on platelets, the key players in thrombogenesis, demand serous investigation.

Proteasome, the protein degradation machinery of the cell, cleaves intracellular proteins in order to regulate essential cellular processes like antigen processing, cell cycle, transcription and signal transduction [33]–[36]. We have recently demonstrated a central role of proteasome in determination of platelet life span [37] and the factors regulating its enzymatic activity in human platelets [38]. In the present study, we have evaluated effect of aspirin on platelet survival in murine as well as human models. Aspirin induced apoptosis in human platelets in a dose-dependent manner, which was associated with concomitant inhibition of platelet proteasomal activity in presence of the drug. Platelet half life was found to be significantly lowered in aspirin-treated mice as compared to control animals. Thus, above observations provide cautionary framework to critically re-evaluate prophylactic and therapeutic dosage regime of aspirin in clinical practice. Despite widespread use of aspirin against cardiovascular ailments, its preventive administration in susceptible individuals on routine basis is not recommended [39]–[41]. Aspirin has been reported to be safe for primary prevention at coronary event risk >1.5%/year though it is not recommended due to associated side effects at coronary event risk 0.5%/year [42]. In standard doses (325 mg) aspirin poses less threat of gastrointestinal bleeding; however, the bleeding risk is still twice as high as without aspirin [43].

### Ethics Statement

The animal study was approved by the Central Animal Ethical Committee of Institute of Medical Sciences, Banaras Hindu University, Varanasi. Swiss Albino mice weighing between 18 and 20 gm (20 control and 40 aspirin-treated) were used in the entire study. All efforts were made to minimize the number of animals used and their suffering.

### Materials and Methods

ABT-737 was purchased from Selleck Chemicals. Anti-CD61-PE and annexin V-PE were from BD Pharmingen. Proteasome inhibitor PSI [Z-Ile-Glu(OtBu)-Ala-Leu-CHO] procured from Calbiochem. N-hydroxysuccinimidobiotin (NHS-biotin), PE-strepatvidin, 5,5′-6,6′-tetrachloro-1,1′,3,3′ tetraethylbenzimidazolylcarbocyanine iodide (JC-1), thiazole orange, carbonyl cyanide 3-chlorophenylhydrazone (CCCP), 6-carboxy-2′,7′- dichlorodihydrofluorescein (H_2_DCFDA), acetyl-Asp-Glu-Val-Asp-7-amido-4-methylcoumarin (AC-DEVD-AMC), apyrase, ethylene glycol tetra acetic acid (EGTA), ethylene diamine tetra acetic acid (EDTA), sodium orthovanadate, acetylsalicylic acid (aspirin), bovine serum albumin (fraction V), Triton X-100, protease inhibitors, mouse monoclonal anti-Bax (6A7) were purchased from Sigma. RPMI 1640 was purchased from HiMedia. Reagents for electrophoresis were products of Merck. PVDF membranes were from Millipore. SuperSignal West Pico chemiluminescent substrate was from Pierce. Horseradish peroxidase (HRP)-labeled secondary antibodies were purchased from Transduction Laboratories and Bangalore Genei, respectively. All other reagents were of analytical grade. Type 1 deionized water (18.2 MΩ.cm, Millipore) was used for preparation of solutions.

### Platelet preparation

Platelets were isolated from fresh human blood by differential centrifugation, as already described [44]. Briefly, blood was collected from healthy volunteers (majority male) under informed consent and centrifuged at 180×g for 10 min. PRP (platelet-rich plasma) thus obtained was incubated with 1 mM acetylsalicylic acid for 15 min at 37°C. After addition of EDTA (ethylenediaminetetraacetic acid) (5 mM), platelets were sedimented by centrifugation at 800×g for 15 min. Cells were washed in buffer A (20 mM HEPES, 138 mM NaCl, 2.9 mM KCl, 1 mM MgCl_2_, 0.36 mM NaH_2_PO_4_, 1 mM EGTA (ethylene glycol tetraacetic acid), supplemented with 5 mM glucose and 0.6 ADPase units of apyrase/ml, pH 6.2) and were finally resuspended in buffer B (20 mM HEPES, 138 mM NaCl, 2.9 mM KCl, 1 mM MgCl_2_, 0.36 mM NaH_2_PO_4_, pH 7.4). The final cell count was adjusted to 0.5−.8×10^9^/ml. All steps were carried out under sterile conditions and precautions were taken to maintain the cells in resting condition.

### Proteasome activity assay

Platelets were incubated at 37°C for 30 min with aspirin (2, 5 and 10 mM) or vehicle (ethanol) and washed twice in buffer A (see above). Cells were pelleted and resuspended in 125 µl of 2× permeabilization buffer (20 mM HEPES, 0.2% Triton X-100, 300 mM NaCl and 2 mM EGTA, pH 7.7) followed by addition of 125 µl of 2× proteasome assay buffer (40 mM HEPES, 1 mM EDTA and 0.07% SDS, pH 7.8). Permeabilized cells were added to the wells of microplates in a fluorescence microplate reader (BioTek model FLx800) at 37°C. Reaction was started by the addition of Suc-Leu-Leu-Val-Tyr-AMC (25 µM) in DMSO and was monitored for 10 min (excitation, 360 nm; emission, 460 nm) [37]. Proteasome peptidase activities were determined from Suc-LLVY-AMC-hydrolyzing activity (chymotrypsin-like activity).

### Cytofluorimetric analysis of mitochondrial transmembrane potential

Mitochondrial transmembrane potential (Δψ) was measured using the potential-sensitive fluorochrome JC-1, which selectively moves across polarized mitochondrial membrane and forms aggregates (red). As membrane potential collapses, color changes from red to green due to release of monomeric dye [37]. In order to study Δψ, platelets were pre-treated with aspirin (2, 5 and 10 mM) or ethanol (vehicle) for 30 min, followed by incubation with 2 µM JC-1 for 15 min at 37° C in dark. Cells were washed in phosphate-buffered saline (PBS) and JC-1 fluorescence was analyzed in FL1 and FL2 channels of flow cytometer (FACSCalibur, Becton Dickinson) for detection of dye monomer and aggregates, respectively. The ratio of red to green (FL2/FL1) fluorescence reflected mitochondrial transmembrane potential.

### Flow cytometric measurement of reactive oxygen species (ROS)

Platelets were treated with aspirin (2, 5 and 10 mM) or ethanol (vehicle) as above, washed with PBS and incubated with H_2_DCFDA (1 µM) for 30 min at 37°C in dark. Cells were next washed twice with PBS and analyzed by flow cytometry as described previously [45].

### Measurement of annexin v and P-selectin binding by flow cytometry

Platelets (1×10^8^ cells in 100 µl) were incubated at 37°C for 30 min in the presence aspirin or vehicle as stated above. For positive control the washed platelets were treated with thrombin (1 U/ml) for 10 min without stirring. Then equal amount of 4% paraformaldehyde was added to the cells and incubated for 30 min, which was followed by washing. Post-fixed resuspended platelets were labeled with 5 µl FITC-labeled P-selectin antibody, 5 µl PE-labeled anti-CD61 antibody and 10 µl FITC-labeled annexin V (in preseance of 5 mM CaCl_2_ to promote binding). Samples were incubated for 30 min at RT in dark and analyzed on the flow cytometer [45]. After compensation between FITC and PE, all fluorescence data were collected using four-quadrant logarithmic amplification. Data from CD61-positive 10,000 events were collected for each sample.

### Caspase-3 activity assay

To determine cytosolic caspase-3 activity, samples were pre-treated with 2, 5 and 10 mM of aspirin or vehicle (ethanol) and lysed with equal amount of 2× RIPA buffer. After 10 min incubation in ice, equal volume of 2× substrate buffer (20 mM HEPES, pH 7.4, 2 mM EDTA, 0.1% CHAPS, 5 mM DTT and 10 µM caspase substrate AC-DEVD-AMC) was added to each lysate and further incubated for 30 min at 37°C [45]. Caspase-3 activity was determined from the extent of cleavage of fluorogenic substrate measured at 460 nm emission (excitation, 360 nm).

### Western blotting

Proteins were separated by 13% SDS-PAGE and electrophoretically transferred onto PVDF membrane (0.8 mA/cm^2^, 2 h) in a semi-dry blotter (TE 77 PWR, GE Healthcare) for subsequent probing as described previously [37]. Blots were incubated for 1 hr with 5% (w/v) BSA in Tris-buffered saline containing 0.05% Tween 20 (TBST) to block residual protein binding sites. Membranes were incubated overnight at 4°C with the primary antibody (anti-Bax, 1∶500). Blots were incubated with the appropriate HRP-conjugated secondary antibody (diluted 1∶10,000) and exposed to enhanced chemiluminescence reagents for 5 min. Blots were exposed to photographic films and densitometrically scanned. For protein loading control, membranes containing whole cell lysates were reprobed with the anti-actin antibody (1∶1000).

### Platelet clearance analysis

Mice were orally administered with aspirin (10 and 15 mg/kg/day, respectively, in two groups) for 4 days. The control animals were administered with vehicle. In different experiments mice were intravenously injected with 50 and 75 mg/kg of aspirin. NHS-biotin (600 mg) was injected in tail vein of either ethanol (control) or aspirin administered mice [46]. At various time points 50 µl retro-orbital blood was drawn from both control as well as treated mice, mixed with 200 µl buffered saline-glucose-citrate buffer (116 mM NaCl, 13.6 mM trisodium citrate, 8.6 mM Na_2_HPO_4_, 1.6 mM KH_2_PO_4_, 0.9 mM EDTA, 11.1 mM glucose) and followed by 1 ml balanced salt solution (149 mM NaCl, 3.7 mM KCl, 2.5 mM CaCl_2_, 1.2 mM MgSO_4_, 7.4 mM HEPES, 1.2 mM KH_2_PO_4_, 0.8 mM K_2_HPO_4_, 3% bovine calf serum). Cells were pelleted at 1400×g for 10 min, and resuspended in 300 µl sheath fluid. They were stained with FITC-conjugated rat anti-CD41, which label only platelets, followed by PE-streptavidin for 1 h on ice, washed in balanced salt solution and analyzed by flow cytometry to determine the fraction of platelet population labeled with PE.

### Monocyte isolation, culture and phagocytic recognition of platelets

Human monocytes were isolated and cultured as described [47], [48]. Briefly, blood from healthy donors was collected in citrate and peripheral blood mononuclear cells (PBMCs) were isolated using Hysep, according to manufacturer's instructions. Monocytes were further isolated by plating the PBMCs on polystyrene-coated tissue culture flasks for 4 h at 37°C, followed by 3 washes with PBS to remove non-adherent lymphocytes. Monocytes (250,000 in 500 µl volume) were then plated on 6-well plates in RPMI 1640 supplemented with 10% fetal bovine serum and cultured for 7 days to obtain the monocyte-derived macrophages (MDMs). Platelets labeled with calcein-AM were incubated with a monolayer of autologous monocyte-derived adherent human macrophages for 45 min. Following incubation period, the phagocyte monolayer was washed free of non-interacting platelets, and any adherent platelets were removed by treatment with trypsin at 37°C for 5 min followed by 5 mM EDTA at 4°C. MDMs were recovered by trypsin/EDTA treatment for 15 min at 37°C and subjected to flow cytometric and epifluorescent microscopic analysis.

In different experiments, aspirin (10 mg/kg/day) was administered orally to mice for 7 days, following which macrophages were isolated from the peritoneal cavity of animals. Platelets were isolated from control as well as aspirin-administered mice and stained with calcein-AM. Equal number of platelets from either group was co-incubated *ex vivo* with respective pre-isolated macrophages for 45 min in CO_2_ incubator. Macrophages were gated and analyzed by flow cytometry for fluorescence signal (FL1).

### Statistical methods

Standard statistical methods were used. Parametric methods (*t* test) were used for evaluation and tests were considered significant at *P*<0.05 (2-tailed tests). Data are presented as means ± SD of at least five individual experiments from different blood donors.

## Results

### Aspirin induces apoptosis-like phenotype in human platelets associated with upregulation of Bax and proteasomal inhibition

**Figure 1:**
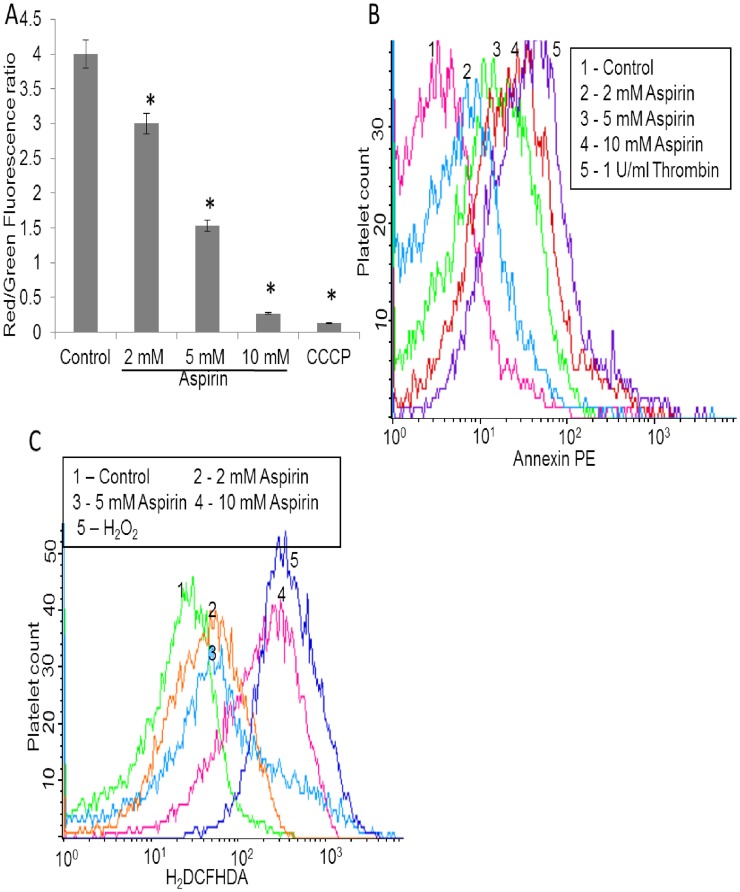
Study of apoptosis-like features in platelets following aspirin treatment. Mitochondrial transmembrane potential (red/green ratio) (A), PS exposure (PE-annexin V binding) (B) and ROS generation (C) were studied in control platelets, as well as in cells pre-treated with aspirin as indicated. In (A), CCCP (mitochondrial protonophore) has been employed as the positive control. Data are representative of five different experiments and expressed as mean±SD. (*p<0.05 as compared to ethanol-pretreated resting platelets).

NSAIDs have been reported to induce apoptosis presumably independent of their ability to inhibit cyclooxygenase activity [5]–[8]. In order to evaluate aspirin action on platelets, we investigated mitochondrial transmembrane potential (Δψ_m_) and surface exposure of phosphatidylserine (PS) in aspirin-treated human platelets. JC-1, a lipophilic cation, was employed to determine alterations in Δψ_m_. As expected, high aggregate to monomer fluorescence ratio (FL2/FL1) of the fluor was observed in untreated (control) cells indicative of stabilized Δψ_m_, which dropped drastically in carbonyl cyanide 3-chlorophenylhydrazone (CCCP) (protonophore) (30 µM)-treated platelets (Fig. 1A). Incubation of platelets with different concentrations of aspirin (2, 5 and 10 mM) was associated with significant decrements in Δψ_m_ (by 25%, 61% and 93%, respectively) as compared to control (Fig. 1A).

Externalization of PS, an anionic phospholipid, from the inner leaflet to outer layer of cell membrane is an invariable feature in the apoptotic process [37], [49]. PE-annexin V binding to cell surface was studied as a measure of cells undergoing apoptosis. As expected, surface membrane was found to be significantly enriched with PS in thrombin-stimulated platelets as compared to the resting cells. Remarkably, pretreatment of platelets with increasing concentrations of aspirin (2, 5 and 10 mM) resulted in dramatic increase (by 1.69-, 7- and 11-folds, respectively) in annexin V binding as compared to the untreated (control) cells, indicative of induction of apoptosis-like changes upon aspirin treatment (Fig. 1B). Salicylic acid (10 mM) did not elicit increment in annexin V binding, which indicated that aspirin caused PS exposure by acetylation-dependent mechanism. To rule out the possibility of platelet activation in response to different dosages of aspirin, we examined surface expression of P-selectin, a marker for platelet activation, in aspirin-treated platelets. Aspirin had no effect on surface level of P-selectin, which was found to be significantly elevated on surface membrane in thrombin-stimulated platelets (Figure S1).

As apoptosis is known to be associated with cellular reactive oxygen species (ROS) generation [50], we sought to determine whether aspirin induced ROS production in platelets. Using the cell-permeable dye 2′,7′-dichlorofluorescein diacetate (H_2_DCFDA), intracellular ROS was found to be significantly enhanced (by 1.96-, 6- and 8- folds) in platelets treated with 2, 5 and 10 mM of aspirin, respectively (Fig. 1C).

**Figure 2:**
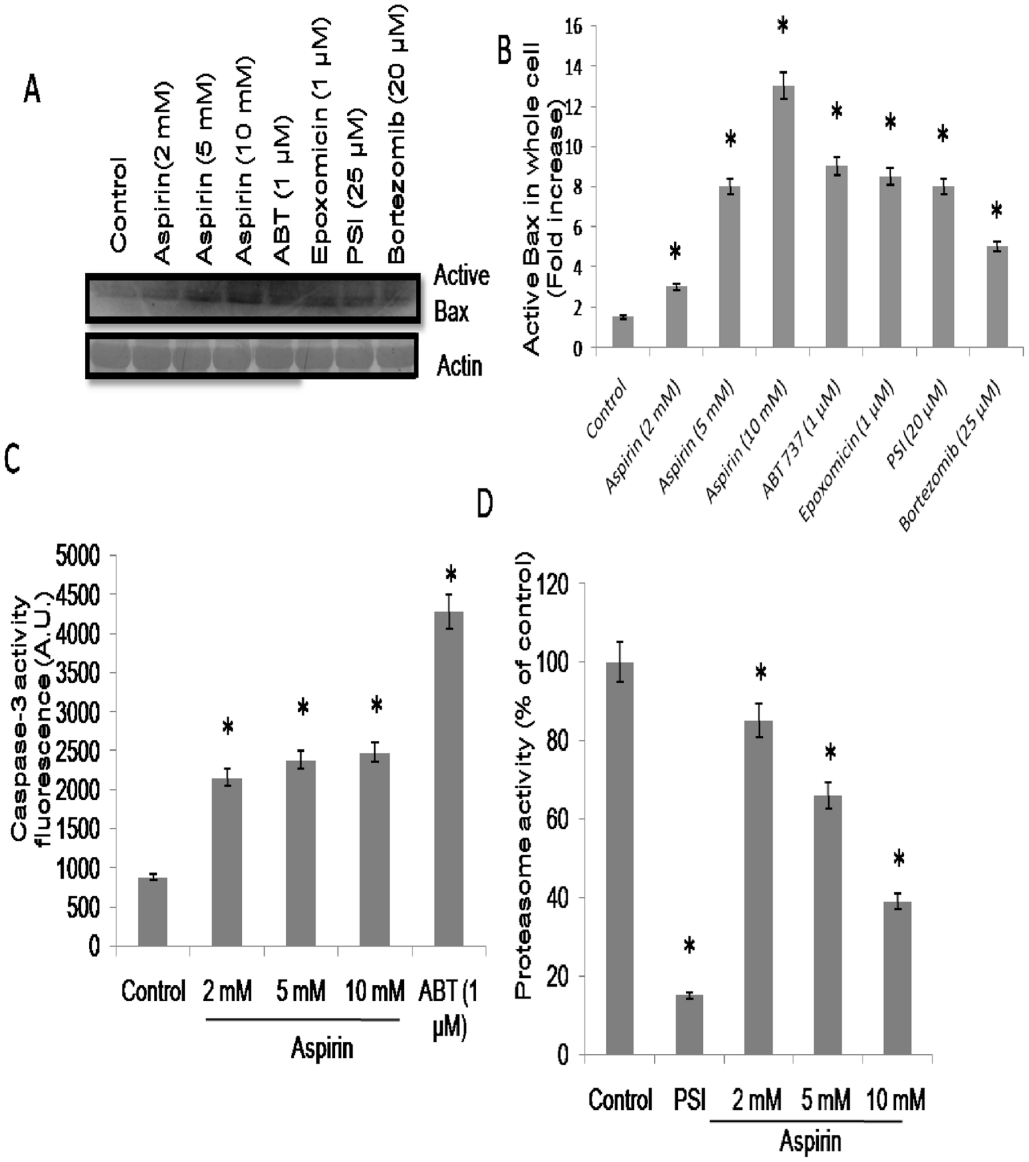
Study of proteosome and caspase-3 activities in aspirin-treated platelets (A), Western blots showing expression level of active Bax in platelets pretreated with ethanol, aspirin, ABT737, epoxomicin, PSI and bortezomib, as indicated (upper panel) normalized against β-actin (lower panel). (B), Quantitative representation of active Bax levels in platelet whole cell lysates determined by densitometry of Western blots. (C), caspase-3 activity from the extent of cleavage of fluorigenic substrate AC-DEVD-AMC. (D), Assay of proteasome enzymatic activity in platelets pretreated with ethanol, PSI (proteasome inhibitor) (10 µM) and aspirin. Data are representative of five different experiments and expressed as mean±SD. (*p<0.05 as compared to ethanol-pretreated resting platelets).

Early in the process of apoptosis Bax, the pro-apoptotic member of Bcl-2 family, is known to undergo conformational change, followed by translocation to the mitochondrial membrane [51]. We evaluated level of conformationally-changed active Bax in aspirin pre-treated platelets using an antibody that specifically recognizes Bax in its conformationally altered state (clone 6A7). Exposure of platelets to aspirin in increasing concentrations (2, 5 and 10 mM) resulted in progressive increments in level of conformationally active Bax (by 2-, 6- and 11- folds, respectively) (Fig. 2A & B). BH3-mimetic ABT737 and specific proteasome inhibitors like epoxomicin (1 µM), PSI (20 µM) and bortezomib (25 µM) were used as a positive control, which induced significant Bax activation in platelets (Fig. 2A & B).

The pro-apoptotic members of Bcl-2 family induce release of mitochondrial cytochrome c into cytosol, which eventually leads to caspase-3 activation through constitution of apoptosome complex with Apaf-1 and caspase-9 [48]. As reported earlier [46], ABT737 induced significant activation of caspase-3 in platelets (Fig. 2C). A concentration-dependent upregulation in caspase-3 activity (by 2-, 2.8- and 3- folds) (Fig. 2C) was observed in platelets exposed to increasing doses of aspirin (2, 5 and 10 mM, respectively).

Proteasome is involved in the degradation of many short-lived proteins that are required for cell survival. We have recently demonstrated a central role of proteasome in delimiting platelet life span through constitutive elimination of the conformationally active Bax and inducing apoptosis-like changes in platelets [37]. In order to explore the effect of aspirin on proteasome in human platelets, we analyzed proteasomal peptidase activity in presence of different concentrations of aspirin. We found a concentration-dependent attenuation (by 15%, 44% and 61%) in proteasome function in human platelets pre-treated with aspirin (2, 5 and 10 mM, respectively) (Fig. 2D). Aspirin treatment did not induce any change in the total protein content in platelets.

### Aspirin leads to decreased platelet life span

**Figure 3:**
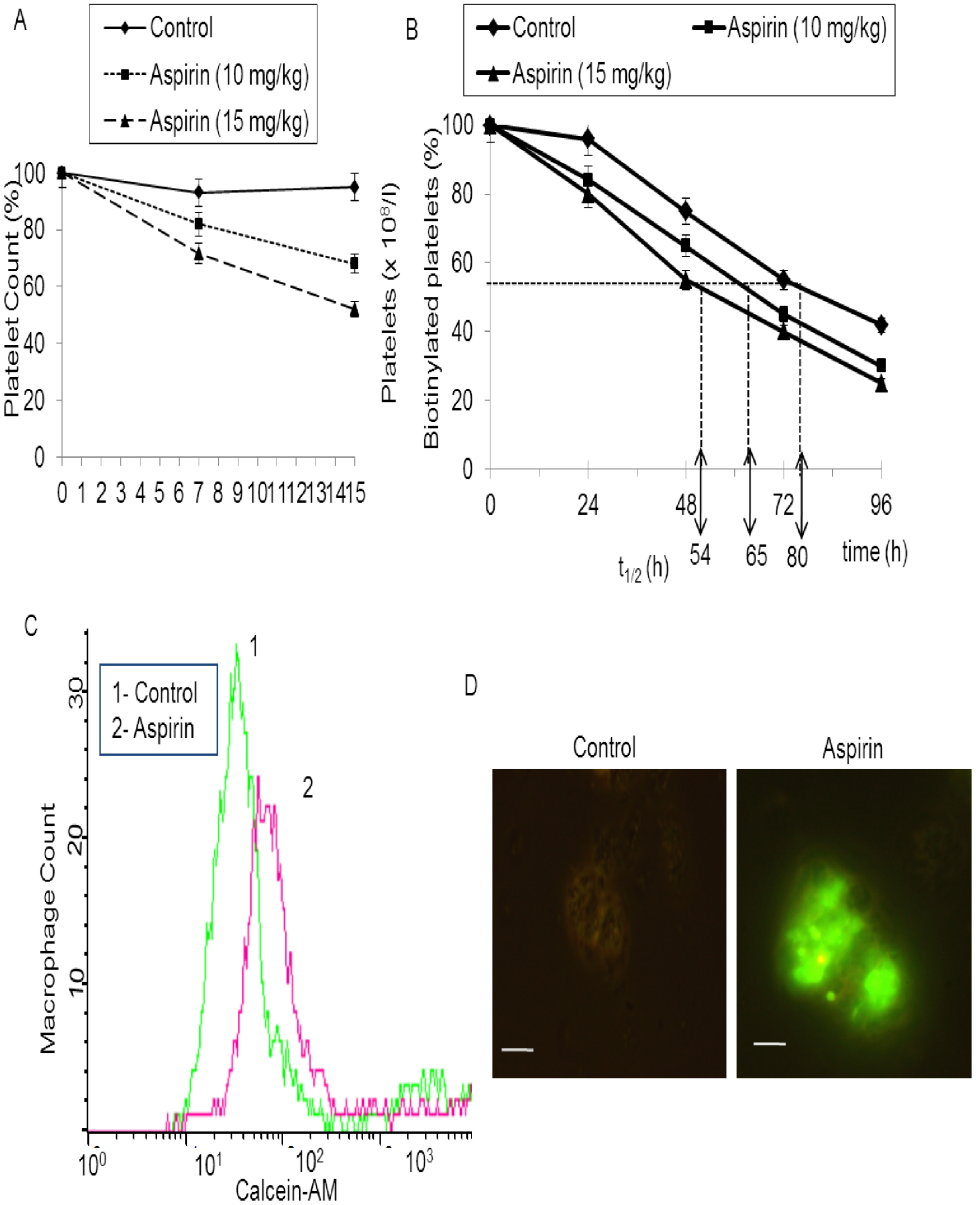
Aspirin affects lifespan and phagocytic uptake of platelets (A), Platelet count in control as well as aspirin-administered mice on different days. (B), Proportion of biotinylated platelets (%) in peripheral blood sample drawn from ethanol (vehicle) or aspirin (10 and 15 mg/kg) pre-administered mice 0, 24, 48, 72, and 96 h after administration of NHS-biotin. t_1/2_ (h) represents platelet half-life in hours. (C) and (D), phagocytic uptake of platelets by autologous macrophages. Flow cytometry (C) and epifluorescence microscopy (D) of macrophages co-incubated with calcein-labeled platelets pretreated either with aspirin (5 mM) or ethanol (control). Scale bars, 10 µm. Data are representative of five different experiments.

Platelet life span is determined by opposing activities of anti- and pro-apoptotic Bcl-2 family proteins [46]. As proteasomal activity in tumor cells influences cellular level of these proteins and aspirin attenuated proteasome function in platelets (our earlier figure), we investigated if aspirin can regulate platelet lifespan *in vivo* in a rodent model. Consistent with earlier findings [30], [31] oral administration of aspirin to mice led to significant lowering in platelet count by 18% and 32% (on 7^th^ and 15^th^ day, respectively, at 10 mg/kg/day) and 28% and 48% (on 7^th^ and 15^th^ day, respectively, at 15 mg/kg/day) (n = 5) (Fig. 3A). When administered intravenously in tail vein in mice, 50 and 75 mg/kg of aspirin decreased platelet count by 50% and 72%, respectively, reflective of aspirin-induced decrease in platelet count (data not shown).

Reduction in platelet count could either be due to increase in platelet clearance or decreased platelet production. In order to study former possibility we conjugated mice platelets with biotin by intravenous administration of NHS-biotin and tracked the labeled platelets *ex vivo* by incubating cells with PE-streptavidin [44]. Consistent with earlier observation by Berger *et al.* (1998), platelet half-life (t_1/2 = _clearance of 50% biotin-conjugated platelets) was found to be 80 h in control mice, which dropped significantly to 65 h and 54 h, respectively, in mice administered orally with aspirin at 10 and 15 mg/kg/day for 4 days (Fig. 3B). Intravenous administration of 50 and 75 mg/kg of aspirin to the tail vein of mice decreased half life of platelets by 21 and 37 h, respectively (data not shown). Salicylic acid (75 mg/kg) did not affect the platelet life span, suggesting that aspirin lowered platelet life span via acetylation-dependent mechanism.

### Aspirin enhances uptake of platelets by macrophages

Platelets undergoing apoptosis-like changes are known to be removed by phagocytes in the reticulo-endothelial system [52]. Hence, we evaluated macrophage-assisted clearance of platelets following aspirin treatment. Calcein-stained platelets, either pretreated with aspirin (5 mM) or ethanol (vehicle), were incubated with a monolayer of autologous monocyte-derived adherent human macrophages for 45 min. The phagocyte monolayer was next washed free of non-interacting platelets, and subjected to flow cytometry as well as epifluorescence microscopy to examine phagocytic uptake of platelets by macrophages.

Macrophages were gated and analyzed by flow cytometry for fluorescence signal (FL1). The cells exhibited significantly higher calcein fluorescence following incubation with aspirin-treated fluorescently labeled platelets, which was reflective of enhanced phagocytic uptake of these platelets (Fig. 3C). This finding was further corroborated from epifluorescence microscopy of macrophages. Significantly higher mean fluorescence intensity (per high power field) was found to be associated with macrophages co-incubated with aspirin-treated platelets than the control cells, which reflected facilitated phagocytic uptake of platelets upon exposure to aspirin (Fig. 3D). In different experiment, platelets from mice administered orally with aspirin (10 mg/kg/day for 7 days) were incubated with macrophages obtained from the same animals. Cells from experimental mice exhibited significantly higher calcein fluorescence than the control counterparts (data not shown), which was reflective of enhanced phagocytic uptake of these cells.

## Discussion

Aspirin is one of the most widely used medications worldwide, with more than 100 billion tablets consumed each year [53]. Aspirin is extensively used under clinical settings as an anti-inflammatory drug and for prevention of thrombus formation/propagation in myocardial infarction as well as stroke by inhibition of platelet COX-1 activity. Apart from above functions, aspirin and other NSAIDs also reportedly exhibit antiproliferative effect. In this study we have investigated apoptosis-like changes in human platelets elicited by aspirin and explored its effect on murine platelet lifespan *in vivo*.

Here we found that, platelets exhibited features of apoptosis following aspirin treatment, which included drop in mitochondrial transmembrane potential, enhanced surface exposure of PS, rise in cytosolic ROS and activation of caspase-3. The pro-apoptotic Bcl-2 family protein Bax and Bax-specific mRNA are known to be abundantly expressed in platelets [54], [55]. Aspirin treatment provoked significantly higher expression of conformationally active Bax in platelets compared to their control (untreated) counterparts. Bax being a known substrate of proteasome [56], [57] we next examined effect of aspirin on proteasomal peptidase activity in order to understand mechanistic underpinning of aspirin effects on platelets. The drug was found to elicit concentration-dependent attenuation in proteasomal function in human platelets (Fig. 2D), which was in line with an earlier study on murine Neuro 2a cells [29]. However, above observations did not formally establish a link between proteasome inhibition and Bax activity. A limitation of this study is high dose of aspirin administered in mice. Although effective therapeutic dosage of aspirin against coronary artery disorders is lower than that employed in this study, drug regime against rheumatoid arthritis [9] and other systemic inflammatory diseases is higher by 5–10 fold, which is fairly comparable with aspirin orally administered in mice in our study. Salicylic acid (10 mM) did not evoke any apoptotic response, which indicated that effect of aspirin could be due to acetylation-dependent process.

We have recently shown that, proteasome plays critical role in platelet survival through constitutive elimination of the conformationally active Bax [37]. As aspirin inhibited proteasome activity in human platelets, we next asked whether the drug would adversely affect platelet life span in an *in vivo* rodent model. Administration of mice with aspirin led to thrombocytopenia. This was associated with significant reduction in half-life of circulating platelets, indicative of enhanced rate of platelet clearance in aspirin-administered mice. Consistent with this, aspirin-treated human platelets were found to be phagocytosed more efficiently by macrophages, as demonstrated *in vitro* by flow cytometry as well as epifluorescence microscopy

To summarize, we have shown that aspirin elicits apoptosis-like changes in human platelets *in vitro* and delimits platelet life span in murine model, which was associated with proteasomal inhibition and increased expression of conformationally active Bax. Our observations support the contention that, platelets should not be collected from donors under NSAID cover for purpose of transfusion. A recent report has implicated platelets with cancer cell proliferation [58] and aspirin has been directly linked to prevention of cancer [59]. Thus, the observed drop in longevity of aspirinized platelets suggests a novel mechanistic insight of therapeutic benefit of this drug in cancer management. Aspirin is widely used as an anti-inflammatory drug against rheumatoid arthritis and towards prevention of occlusive platelet thrombi in coronary as well as cerebral thrombotic events including myocardial infarction. Therefore, observations from this study provide cautionary framework to critically re-evaluate therapeutic dosage regime of aspirin especially against systemic inflammatory ailments though it is a relatively safe drug in general clinical practice.

## Supporting Information

Figure S1 **Study of P-selectin exposure in aspirin treated platelets and thrombin (1 U/ml) was used as positive control.**
(DOCX)
