# Correction: Aspirin Delimits Platelet Life Span by Proteasomal Inhibition

**DOI:** 10.1371/journal.pone.0115927

**Published:** 2014-12-12

**Authors:** The PLOS ONE Staff

The lower panel Western blot in Figure 2A is incorrect. The authors have provided a corrected Figure 2 here.

**Figure 2:**
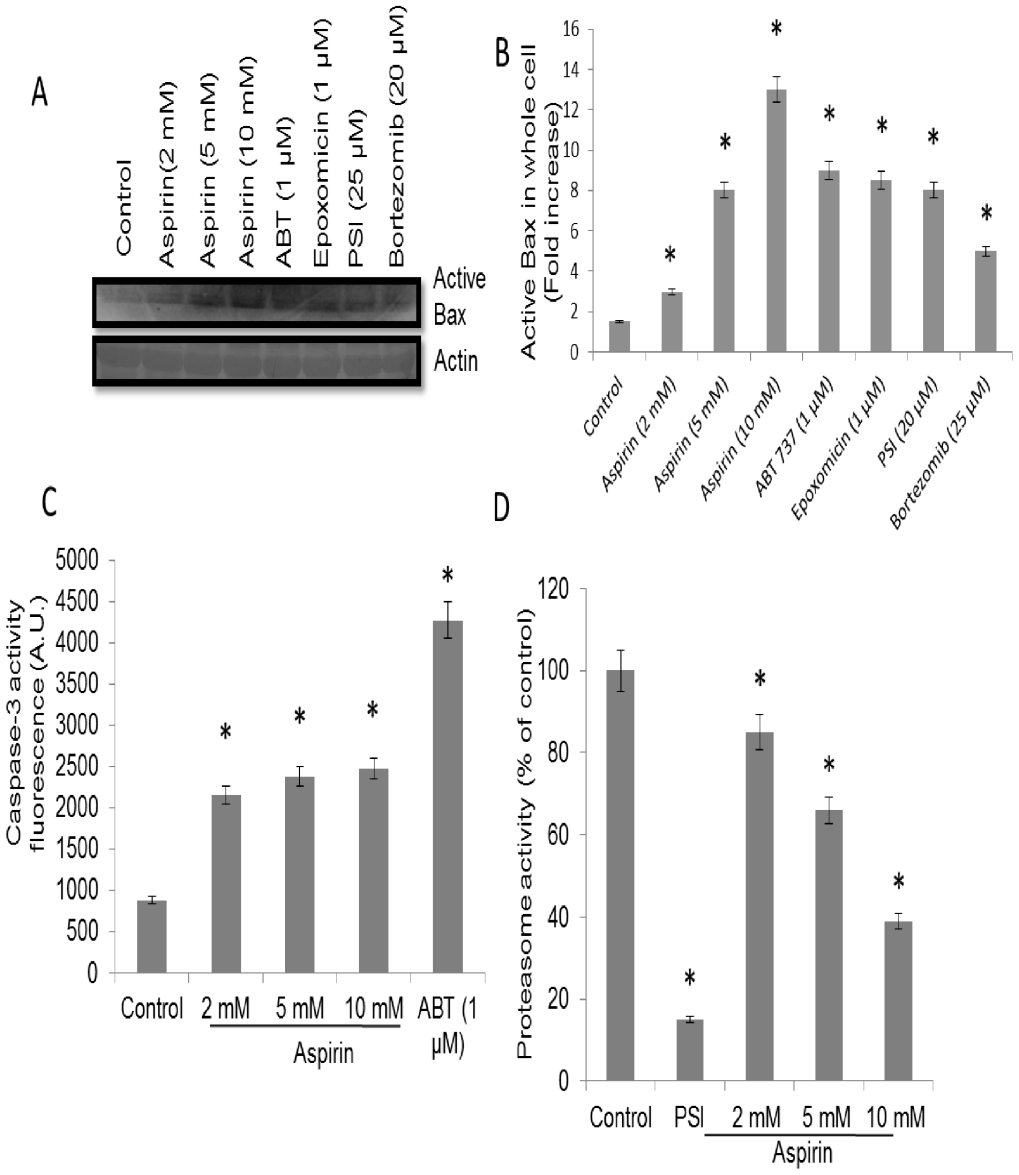
Study of proteosome and caspase-3 activities in aspirin-treated platelets (A), Western blots showing expression level of active Bax in platelets pretreated with ethanol, aspirin, ABT737, epoxomicin, PSI and bortezomib, as indicated (upper panel) normalized against β-actin (lower panel). (B), Quantitative representation of active Bax levels in platelet whole cell lysates determined by densitometry of Western blots. (C), caspase-3 activity from the extent of cleavage of fluorigenic substrate AC-DEVD-AMC. (D), Assay of proteasome enzymatic activity in platelets pretreated with ethanol, PSI (proteasome inhibitor) (10 µM) and aspirin. Data are representative of five different experiments and expressed as mean±SD. (*p<0.05 as compared to ethanol-pretreated resting platelets).
