# Supplementary material for: Aspirin Delimits Platelet Life Span by Proteasomal Inhibition
Source: PLoS One. 2014 Aug 15;9(8):e105049. doi: 10.1371/journal.pone.0105049 (PMC4134270; doi:10.1371/journal.pone.0105049)
Supplement: Figure S1 — Study of P-selectin exposure in aspirin treated platelets and thrombin (1 U/ml) was used as positive control. (DOCX) [file pone.0105049.s001.docx]

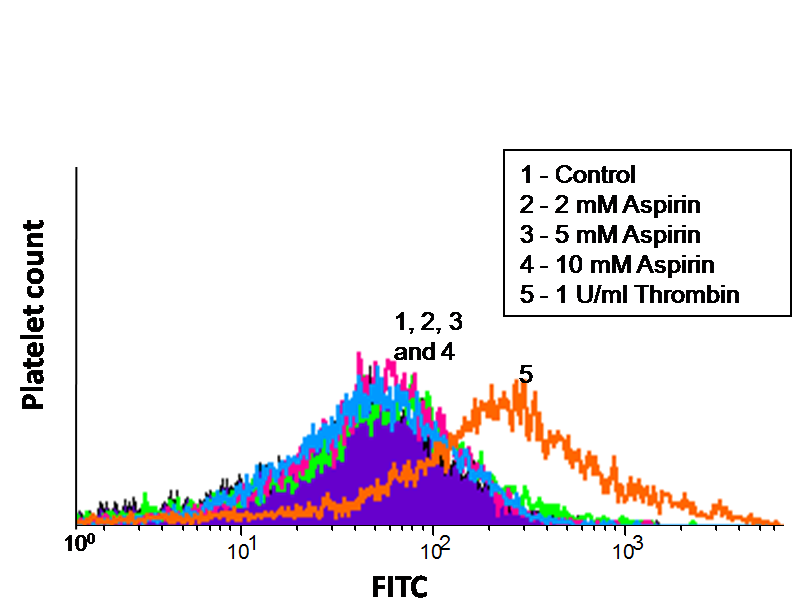


**Figure S1.** Study of P-selectin exposure in control, as well as aspirin (2, 5 and 10 mM)-treated platelets. Thrombin (1 U/ml) was used as positive control. Data are representative of five different experiments.
